# Obstructive Sleep Apnea Susceptibility Genes in Chinese Population: A Field Synopsis and Meta-Analysis of Genetic Association Studies

**DOI:** 10.1371/journal.pone.0135942

**Published:** 2015-08-18

**Authors:** Jinxian Sun, Jianrong Hu, Chunlin Tu, Anyuan Zhong, Huajun Xu

**Affiliations:** 1 Department of Respiratory Medicine, Jiading Central Hospital, 1 Chengbei road, Shanghai, 201800, China; 2 Department of Respiratory Diseases, the Second Affiliated Hospital of Soochow University, 1055 Sanxiang Road, Suzhou, 215004 China; 3 Department of Otolaryngology, Shanghai Jiao Tong University Affiliated Sixth People’s Hospital, Otolaryngology Institute of Shanghai Jiao Tong University, 600 Yishan Road, Shanghai, 200233, China; Weill Cornell Medical College in Qatar, QATAR

## Abstract

**Background:**

Epidemiological studies to date have evaluated the association between genetic variants and the susceptibility to obstructive sleep apnea (OSA). However, the results of these studies have been inconclusive. In this current study we performed meta-analysis of genetic association studies (GAS) to pool OSA-susceptible genes in Chinese population, to perform a more precise evaluation of the association.

**Methods:**

Various databases (i.e., PubMed, EMBASE, HuGE Navigator, Wanfang and CNKI) were searched to identify all eligible GAS-related variants associated with susceptibility to OSA. The generalized odds ratio metric (OR_G_) and the odds ratio (OR) of the allele contrast were used to quantify the impact of genetic variants on the risk of OSA. Cumulative and recursive cumulative meta-analyses (CMA) were also performed to investigate the trend and stability of effect sizes as evidence was accumulated.

**Results:**

Thirty-two GAS evaluating 13 polymorphisms in 10 genes were included in our meta-analysis. Significant associations were derived for four polymorphisms either for the allele contrast or for the OR_G_. The variants TNF-α-308G/A, 5-HTTLPR, 5-HTTVNTR, and APOE showed marginal significance for OR_G_ (95% confidence interval [CI]): 2.01(1.31–3.07); 1.31(1.09–1.58); 1.85(1.16–2.95); 1.79(1.10–2.92); and 1.79(1.10–2.92) respectively. In addition, the TNF-α-308G/A, 5-HTTLPR, and 5-HTTVNTR variants showed significance for the allele contrast: 2.15(1.39–3.31); 2.26(1.58–3.24); 1.32(1.12–1.55); and 1.86(1.12–3.08) respectively. CMA showed a trend towards an association, and recursive CMA indicated that more evidence was needed to determine whether this was significant.

**Conclusions:**

TNF-α, 5-HTT, and APOE genes can all be proposed as OSA-susceptibility genes in Chinese population. Genome-wide association studies (GWAS) are therefore urgently needed to confirm our findings within a larger sample of OSA patients in China.

## Introduction

Obstructive sleep apnea (OSA) is one of the most common sleep breathing disorders, affecting approximately 2% of females and 4% of males in Western countries [1]. In Asia, the prevalence of OSA has been estimated to be from 3.7% to 97.3% [2]. In comparison with Westerners, Chinese are prone to ignoring the means of prevention, diagnosis and treatment of OSA due to inadequate medical resources and poverty. Since there has been a consistent growth in the social and economic burdens caused by OSA, as OSA could bring a range of sequelae (i.e., poor glycemic control and hypertension)[3–5], the establishment of an approach to reduce the risk of OSA is urgently needed in the most heavily populated country in the world.

It is generally believed that OSA is a complex disorder involving multiple traits, and incorporating a hereditary component [6,7]. Both genetic factors and environmental exposures can contribute to the development of OSA [7]. Several meta-analyses have explored the relationship between sporadic genetic variants and risk of OSA [8–13]. However, most of the aforementioned meta-analyses lacked the inclusion of a subgroup analysis stratified by race. As is already known, OSA-susceptible genes may differ among ethnicities, such as, compared with white Europeans, severely obese south Asians had significantly greater prevalence[14].

To date, host genetic susceptibility to OSA has been investigated extensively within Chinese population. However, these sporadic, inconsistent, and small-sample-size studies have limited powers to demonstrate such a relationship. Indeed, some of the results from these studies appear to be incompatible or contradictory. So far, no systematic review and meta-analysis of genetic association studies (GAS) covering all tested polymorphisms and focusing on OSA in Chinese populations has been published. To shed some light on this issue, we reviewed the literature and conducted the Human Genome Epidemiology (HuGE) meta-analysis, including cumulative and recursive cumulative meta-analyses (CMA) following the work of Zintzaras [15,16], to identify all candidate genes associated with susceptibility to OSA in Chinese population.

## Materials and Methods

This meta-analysis followed the Preferred Reporting Items for Systematic Reviews and Meta-Analyses (PRISMA) Checklist [17] and Meta-analysis on Genetic Association Studies Checklist (S1 Checklist).

### Literature search

We performed a search of the literature to identify all studies that evaluated the association between genetic variants and the risk of OSA in Chinese population, using the following electronic databases: PubMed, Excerpta Medica Database (EMBASE), HuGE Navigator, Wanfang and Chinese National Knowledge Infrastructure (CNKI). The search terms we used were as follows: “obstructive sleep apnea hypopnea syndrome” or “OSAHS” or “obstructive sleep apnea syndrome” or “OSAS” or “obstructive sleep apnea” or “OSA” or “sleep apnea”, in combination with “polymorphism” or “variant” or “mutation”. In addition, since high-throughput platforms used for investigating genetic variants have been developed, “genome wide association study” or “GWAS” was also searched. No language restriction was applied. The references of all studies included in the search were also checked to yield further eligible studies. If more than one study reported on a particular population, only the latest or the most complete study was included. Additionally, when a study reported different subpopulation results, we identified each subpopulation as a separate study.

### Selection criteria

The included studies met the following criteria: 1) they should evaluate the association between genetic variants and risk of OSA in Chinese population; 2) the gene polymorphism pooled for meta-analysis should be evaluated in at least two studies; 3) a case-control study design should be used; 4) there should be enough genotype distribution or sufficient data for estimating the generalized odds ratio metric (OR_G_) along with a 95% confidence interval (CI). Alongside this, the following exclusion criteria were used: 1) references, case-reports, abstracts and reviews; 2) the study did not provide detailed genotype data; 3) gene polymorphism pooled for meta-analysis was reviewed in fewer than two studies; 4) a case-control design was not used; and 5) overlapping or duplicate publications. Two reviewers (Drs. Sun and Hu) performed the search and selection process independently of each other.

### Data extraction

Two investigators (Drs. Sun and Hu) independently extracted data from each included study into a standard table. The extracted information included the first author’s surname, year of publication, age of OSA patients, genotyping method and genotype frequencies. Where disagreements occurred, Drs Xu, Zhong and Tu participated, and divergences of opinion were then resolved by a group discussion to reach a final consensus. The authors were contacted through Email if there were queries regarding the studies.

### Statistical analysis

For this meta-analysis, all statistical analyses were performed using STATA (ver. 11.0, Stata Corporation, College Station, TX, USA) and ORGGASMA software (available at http://biomath.med.uth.gr)[18]. The concrete algorithm of OR_G_ and instructions how to operate the ORGGASMA software was provided by Zintzaras[18]. A meta-analysis of GAS was performed on the basis of the allele contrast (risk allele vs. non-risk) [19] and for outcomes reported in >1 study [20]. We used the OR_G_ and 95% CIs through the random effect (RE) model to assess the association between genetic variants and the risk of OSA. The RE model assumes a genuine diversity in the results of various studies [15]. The OR_G_ provides a model-free approach for evaluating the genetic risk in GAS [18]. The Hardy-Weinberg equilibrium (HWE) of genetic frequency distributions for the controls was calculated using Pearson’s χ^2^ method [21]. If the control subjects were not in HWE, an adjusted OR_G_ and 95% CIs was used [22]. Homogeneity was tested using the Q statistic and I^2^ statistic. A *p*-value less than 0.1 indicated the existence of between-study heterogeneity. Subgroup analysis by ethnicity (China vs. other) was performed if data permitted. Harbord’s test was performed to examine the existence of the differential magnitude of effect in large versus small studies in meta-analyses involving at least four studies [23]. A sensitivity analysis was performed to evaluate the stability of results after correction of control subjects deviating from HWE [24]. The mode of inheritance in genetic association was estimated using the degree of dominance index (h index) [25,26]. The h-index is defined as the ratio of the natural logarithms of the ORs of the two orthogonal contrasts (models); i.e., the co-dominant and the additive models [25]. CMA and recursive CMA were carried out to evaluate the trend of the risk effect (i.e. OR) of the allele contrast over time [15]. The CMA indicates the trend in the estimated risk effect and the recursive CMA indicates the stability in risk effect. A *p*-value of less than 0.05 was deemed to indicate statistical significance, unless otherwise stated.

## Results

### Selection process and characteristics of OSA-susceptible GAS

**Fig 1:**
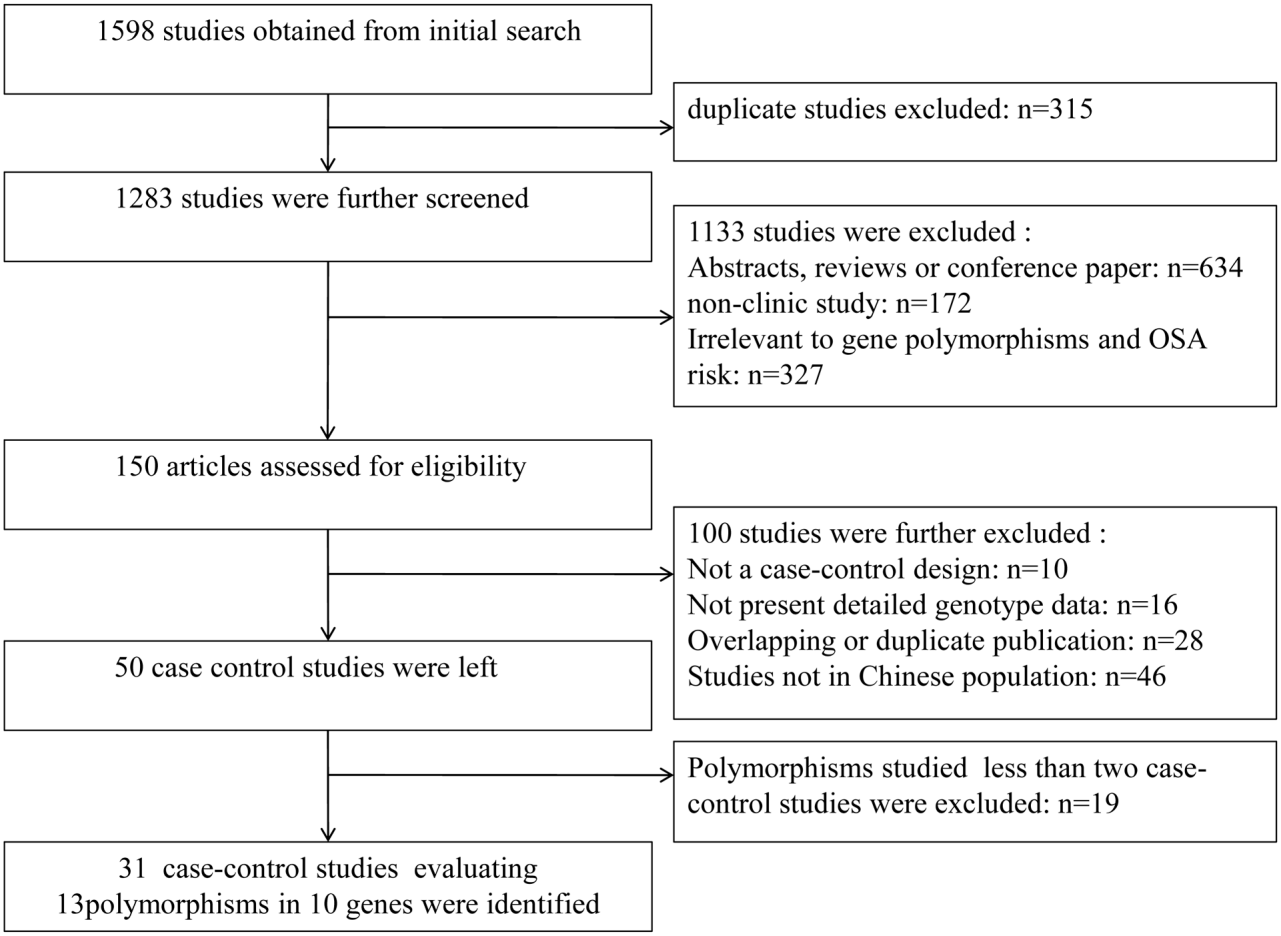
Flow chart of literature search and study selection, with the specification of reasons.

After searching the four electronic databases described above, a total of 1598 references were initially identified. 315 papers were first removed due to duplication. After reading the titles and abstracts, 1133 articles were further excluded for 1) abstracts or reviews, 2) non-clinical studies, and 3) non-relevance to genetic variants and OSA risk. The remaining 150 articles were further screened after reading the full text. Of these, 100 articles were then excluded because they lacked enough data or did not use a case-control design or were not relevant to genetic polymorphisms and OSA risk in Chinese population. Amongst the remaining 50 case-control studies, 19 were further excluded because gene polymorphisms were studied in less than two case-control articles. Finally, a total of 31 case-control studies evaluating genetic variants and OSA risk, involving 13 polymorphisms in 10 genes in Chinese population were identified for quantitative analysis. These articles were published between 1999 and 2014. Fig 1 presents a flow chart of the study inclusion and exclusion criteria.

All polymorphisms were identified by polymerase chain reaction (PCR) (25 studies) or PCR-restriction fragment length polymorphism (PCR-RFLP) techniques (6 studies). Of the 31 case-control studies, 9 polymorphisms were not in HWE, only one study handled with minority population-Uygur [27]. The detail information of each included study is presented in S1 table. The concrete characteristics of each polymorphism are listed in S2–S14 tables.

### Main results, sensitivity analysis and publication bias

In summary, for outcomes >1 available study, a meta-analysis was performed for polymorphisms such as angiotensin-converting enzyme insertion/deletion (ACE I/D)[28–35], tumor necrosis factor (TNF)-α-308G/A[27,36–39], interleukin (IL)-6[40,41], 5-hydroxytryptamine receptor (5-HTR) 2A-102T/C[42–44] and -1438G/A[42–45], 5-HTR2C-796 C/G[42,44], 5-hydroxytryptamine transporter gene-linked promoter region (5-HTTLPR)[36,45–47], 5-HTT variable number tandem repeat (5-HTTVNTR)[44–47], leptin receptor (LEPR) Gln223Arg[48–50], peroxisome proliferator-activated receptor (PPAR-γ) Pro12Ala[51,52], apolipoprotein E (APOE) ε2/ε4[53,54], β1-adrenergic receptor (ADRB1)[55,56] and ADRB2[55,57]. Candidate genes identified from included studies were summarized in Table 1.

**Table 1:** Summary of candidate genes identified from included studies.

Gene	Chromosome location	Polymorphism	Amino acid change	Cases	Controls
ACE	17q23	I/D	NR	580	438
TNF-α	6p21.1–21.3	-308A/G	NR	864	430
IL-6	7p21-14	-572G/C	NR	451	175
5-HTR2A	13q14-q21	-102T/C	NR	396	264
		-1438G/A	NR	489	379
5-HTR2C	q24	-796 C/G	NR	33	28
5-HTT	17q11.1–17q12	LPR	NR	596	756
		VNTR	NR	572	708
LEPR	7q31.3	NR	Gln223Arg	346	339
PPAR-γ	3p25	NR	Pro12Ala	520	290
APOE	19q13.2	ε2/ε3/ε4	NR	436	562
ADRB	5q33.1	NR	Arg389Gly	372	132
		NR	Arg16Gly	345	189

Abbreviation: NR, not reported; ACE, angiotensin-converting enzyme; I/D, insertion/deletion; TNF, tumor necrosis factor; IL-6, Interleukin-6; 5-HTR, 5-hydroxytryptamine receptor; 5-HTT, 5-hydroxytryptamine transporter; LPR, linked promoter region; VNTR, variable number tandem repeat; PPAR-γ, peroxisome proliferator-activated receptor; APOE, apolipoprotein E; ADRB, β-adrenergic receptor.

Table 2 summarizes the associations between various genetic variants and the risk of OSA. The model-free approach and the allele contrast were used. In summary, we obtained significant results from four polymorphisms [TNF-α-308G/A, 2.01(1.31–3.07); 5-HTTLPR, 1.31(1.09–1.58); 5-HTTVNTR, 1.85(1.16–2.95) and APOE, 1.79(1.10–2.92)] in the model-free approach, and three polymorphisms [TNF-α-308G/A, 2.15(1.39–3.31); 5-HTTLPR, 1.32(1.12–1.55); 5-HTTVNTR, 1.86(1.12–3.08)] in the allele contrast (Table 2). The results indicated that the aforementioned genetic polymorphisms were significantly associated with OSA risk in Chinese populations. With regards to TNF-α-308G/A, when we excluded the Uygur study, the result was not affected in the meta-analysis. Finally, the heterogeneity across the included studies ranged from none (P_Q_ = 0.97; I^2^ = 0%) to large (P_Q_ = < 0.01; I^2^ = 99.9%).

**Table 2:** Summary of comparisons of different genetic models for genetic polymorphisms and OSA risk.

Gene Polymorphism	Effect model	OR_G_(95%CI)	P_Q_	I^2^	P_H_	h index
ACE	All model-free	1.46(0.80–2.66)	0.00	85.1	0.18	0.91
	All allele	1.62(0.89–2.94)	0.00	89.4	NC	NC
TNF-α-308G/A	All model-free	2.01(1.31–3.07)	0.18	36.9	0.66	0.31
	All allele	2.15(1.39–3.31)	0.09	50.8	NC	NC
IL-6-572G/C	All model-free	0.96(0.66–1.40)	0.24	26.9	NC	0.70
	All allele	0.99(0.73–1.34)	0.28	15.9	NC	NC
5-HT2A -102C/T	All model-free	1.12(0.86–1.47)	0.99	0	0.70	0.10
	All allele	0.91(0.73–1.13)	0.99	0	NC	NC
5-HT2A-1438G/A	All model-free	1.63(0.78–3.39)	0.00	93.7	0.73	0.50
	All allele	2.02(0.83–4.91)	0.00	94.7	NC	NC
5-HT2C -796 C/G	All model-free	1.00(0.37–2.69)	0.42	0	NC	5.12
	All allele	0.88(0.35–2.24)	0.97	0	NC	NC
5-HTTLPR	All model-free	1.31(1.09–1.58)	0.85	0	0.62	0.09
	All allele	1.32(1.12–1.55)	0.80	0	NC	NC
5-HTTVNTR	All model-free	1.85(1.16–2.95)	0.01	55.7	0.98	0.41
	All allele	1.86(1.12–3.08)	0.03	66.5	NC	NC
LEPR Gln223Arg	All model-free	1.09(0.74–1.59)	0.73	0	0.45	-0.62
	All allele	0.91(0.64–1.29)	0.75	0	NC	NC
PPAR-γ	All model-free	1.40(0.84–2.31)	0.80	0	0.31	0.51
	All allele	0.06(0.01–2.66)	0.00	99.2	NC	NC
APOE	All model-free	1.79(1.10–2.92)	0.86	0	NC	NC
ADRB1Arg389Gly	All model-free	0.97(0.67–1.42)	0.49	0	NC	-0.26
	All allele	0.95(0.68–1.33)	0.58	0	NC	NC
ADRB2 Arg16Gly	All model-free	0.93(0.68–1.26)	0.37	0	NC	NC
	All allele	1.11(0.83–1.48)	0.43	0	NC	1.24

Abbreviation: NC, not calculated; OR_G_, generalized odds ratio; ACE, angiotensin-converting enzyme; I/D, insertion/deletion; TNF, tumor necrosis factor; IL-6, Interleukin-6; 5-HTR, 5-hydroxytryptamine receptor; 5-HTT, 5-hydroxytryptamine transporter; LPR, linked promoter region; VNTR, variable number tandem repeat; PPAR-γ, peroxisome proliferator-activated receptor; APOE, apolipoprotein E; ADRB, β-adrenergic receptor.

For ACE I/D, TNF-α-308G/A, and 5-HTTLPR polymorphisms, cumulative meta-analyses and recursive cumulative meta-analyses were performed (S1–S9 Figs). The pooled genetic risk effect by CMA remained significant in the entire period covered by the papers studied. However, the slight instability in the pooled OR change was found by recursive CMA. Thus, there is a trend towards association and more evidence is needed therefore to draw a safe conclusion on the significance and magnitude of the size of the effect.

Harbold’s test indicated that there was no differential magnitude of effect in large versus small studies for all genetic polymorphisms (Table 2). After correcting for deviation from HWE, the co-dominant and additive models also produced significant ORs (corrected for deviation from HWE); the mode of inheritance (h index) is shown in Table 2.

## Discussion

The current study used a field synopsis and comprehensive meta-analysis of GAS to reveal associations of candidate genetic variants and OSA-susceptibility in Chinese population. In total, 13 gene polymorphisms in 10 genes were identified. Of them, four gene polymorphisms were significantly associated with the risk of OSA, while the other nine were not. Understanding the hereditary pathophysiologic mechanisms in OSA development is essential in establishing effective screening tests, to implement appropriate preventive and therapeutic approaches to early intervention, and to develop gene therapy strategies [58]. Thus, this HuGE meta-analysis may provide useful information for those with a genetic predisposition towards OSA.

In this meta-analysis, we utilized the OR_G_ metric to quantify the magnitude of associations between genetic polymorphisms and OSA risk. Numerous current meta-analyses of GAS have summarized the various genetic contrasts, including the dominant, recessive, additive, and co-dominant. However, these models are not independently satisfied for choosing a specific genetic model, and if more than two models are significant, interpretations of the relative risk effects in these models are not straightforward [18]. In contrast, the OR_G_ metric could overcome the shortcomings of multiple model testing (i.e., lack of biological justification and non-independency of effects). Thus, OR_G_ is optimal for the meta-analysis of GAS for the following reasons: 1) to provide an integrated method to estimate genetic associations by exploring all available information; 2) to make the interpretation of the results easier; 3) to produce more robust results.

In our meta-analysis, we identified all candidate genes from the following six aspects:

1. The Renin-angiotensin system (RAS): the ACE gene, located on chromosome 17q23, contains 26 exons and 25 introns, is critical in the pathogenesis of OSA. ACE is a key enzyme and plays an important role in converting angiotensin I to angiotensin II [59], and serum ACE activity was found to be increased in patients with OSA [60]. Previous meta-analyses revealed no significant associations between ACE I/D polymorphisms and increased OSA risk [10,59,61]. In consistent with former meta-analyses, we also found no association between ACE I/D in the allele contrast and OSA risk in Chinese population.
2. Inflammatory genes: TNF-α, located on region-chromosome 6p, is an important pro-inflammatory cytokine which is elevated in OSA patients and exerts multiple physiological effects on OSA [11]. IL-6, located on chromosome 7p21-14, is another important mediator of inflammatory responses [62]. Using the HuGE meta-analysis, we found that inflammatory gene-TNF-α was indeed associated with increased risk of OSA. This may be explained by the fact that elevated inflammatory cytokines can aggravate upper airways narrowed by edema of the mucosal layers. With regards to IL-6, only two studies were included, we could not give a robust conclusion.
3. Serotonin systems and serotonin transporter systems: 5-HTR2A and 5-HTR2C genes are located on chromosome 13q14–q21 and the q24 region of chromosome X, respectively [9]. The 5-HTT gene is another focus, located on the 17q11.1–17q12 region of chromosome 17 [9]. Consistent with a previous meta-analysis [9], we found that the 5-HTT gene (including 5-HTTLRP and 5-HTTVNTR) was associated with OSA susceptibility, while 5-HTR2A-102T/C was not. However, our results did not reveal a positive association between the 5-HTR2A 1438G/A polymorphism and risk of OSA in Chinese population. In addition, 5-HTR2C was not associated with an increased risk of OSA.
4. Leptin receptor genes: the leptin receptor gene is mapped to 1p31 and has one long isoform and three short isoforms [63]. Neither of the Gln223Arg and Lys109Arg polymorphisms in the LEPR gene showed a positive association with OSA risk.
5. Lipid metabolism genes: the PPAR-γ gene is located on chromosome 3p25 [51]. We found that the Pro12Ala polymorphism in the PPAR-γ gene was indeed associated with OSA susceptibility in Chinese population. The Apo E gene is located on chromosome 19q13.2, with three common alleles: ε2, ε3 and ε4 [12]. Previous meta-analyses showed that there was no association between the APOE gene and OSA risk [7,12]. However, contrary to this, we found in our meta-analysis that the APOE gene was associated with a risk of OSA in Chinese population.
6. Sympathetic nervous system: ADRB is located on chromosome 3p25, and is essential for adiposity differentiation and lipid storage [51]. In our study, we found that ADRB1 and ADRB2 were not associated with OSA risk. However, the sample size concerning the Chinese population was small, and thus further large-scale studies should be carried out to more clearly reveal any such associations.

Some limitations of this meta-analysis should be considered when explaining our results. First, the meta-analysis was based on unadjusted risk estimates for confounding factors (e.g., sex, age, body mass index, life style) not provided by all of the individual GAS. Thus, the existence of effect modifiers may have produced the large heterogeneity between studies, leading to bias [64]. Second, OSA is likely to result from multiple gene-gene interactions occurring in a suitable environment, and we did not consider the potential confounding factors that might have had an impact on the results of the current meta-analysis. The case-control design of each GAS precludes adjusted analysis for gene-gene/gene-environment interactions, and might have reduced the efficiency of genetic risk estimates [65]. Third, a power analysis showed that, to achieve a power of >80% of detecting a modest genetic risk, a sample size of more than 10,000 subjects is needed [64]. Thus, our HuGE meta-analysis might lack sufficient power to detect the weak genetic risk effects of common variants.

Despite the limitations mentioned above, the present meta-analysis first pooled all the sporadic, inconsistent, and small-sample-size studies and provided a cost-effective and a reasonable approach to evaluating the relationship between all candidate genetic polymorphisms and OSA in Chinese population. Of note, the sensitivity analysis and Harbord’s test suggested that the pooled estimates of allelic and genotypic risks obtained in the present study were stable and robust.

In conclusion, the HuGE meta-analysis indicated that the TNF-α, 5-HTT, and APOE genes were associated with OSA susceptibility in Chinese population. These findings may help physicians to formulate personalized prevention and therapy strategies for OSA in Chinese population. Large-scale genome-wide association studies (GWAS) on OSA are needed to expand our understanding of the genetic background to OSA.

## Supporting Information

S1 Checklist Meta-analysis on Genetic Association Studies Checklist. (DOCX)

S1 Fig Odds ratio estimates with the corresponding 95% confidence interval for the allele contrast of angiotensin-converting enzyme insertion/deletion variant and the risk of obstructive sleep apnea in a random-effects model. (TIF)

S2 Fig Cumulative meta-analysis for the allele contrast of angiotensin-converting enzyme insertion/deletion variant and the risk of obstructive sleep apnea. The pooled odds ratio with the corresponding 95% confidence interval at the end of each year-information step is shown.
(TIF)

S3 Fig Recursive cumulative meta-analysis for the allele contrast of angiotensin-converting enzyme insertion/deletion variant. The relative change in the random effects pooled odds ratio (OR) in each information step (OR in next year/OR in current year) is shown. The *Y* axis represents relative change in OR, and the *X* axis represents OR in next year/current year.
(TIF)

S4 Fig Odds ratio estimates with the corresponding 95% confidence interval for the allele contrast of tumor necrosis factor (TNF)-α-308G/A variant and the risk of Obstructive sleep apnea in a random-effects model. (TIF)

S5 Fig Cumulative meta-analysis for the allele contrast of tumor necrosis factor (TNF)-α-308G/A variant and the risk of obstructive sleep apnea. The pooled odds ratio with the corresponding 95% confidence interval at the end of each year-information step is shown.
(TIF)

S6 Fig Recursive cumulative meta-analysis for the allele contrast of tumor necrosis factor (TNF)-α-308G/A variant. The relative change in the random effects pooled odds ratio (OR) in each information step (OR in next year/OR in current year) is shown. The *Y* axis represents relative change in OR, and the *X* axis represents OR in next year/current year.
(TIF)

S7 Fig Odds ratio estimates with the corresponding 95% confidence interval for the allele contrast of 5-hydroxytryptamine transporter gene-linked promoter region variant and the risk of Obstructive sleep apnea in a random-effects model. (TIF)

S8 Fig Cumulative meta-analysis for the allele contrast of 5-hydroxytryptamine transporter gene-linked promoter region variant and the risk of obstructive sleep apnea. The pooled odds ratio with the corresponding 95% confidence interval at the end of each year-information step is shown.
(TIF)

S9 Fig Recursive cumulative meta-analysis for the allele contrast of 5-hydroxytryptamine transporter gene-linked promoter region variant. The relative change in the random effects pooled odds ratio (OR) in each information step (OR in next year/OR in current year) is shown. The *Y* axis represents relative change in OR, and the *X* axis represents OR in next year/current year.
(TIF)

S1 Table Characteristics of included studies in the meta-analysis (DOC)

S2 Table Main data of all included studies for the I/D polymorphism in ACE gene (DOC)

S3 Table Main data of all included studies for the -308A/G polymorphism in TNF-α gene (DOC)

S4 Table Main data of all included studies for the -572G/C polymorphism in IL-6 gene (DOC)

S5 Table Main data of all included studies for the -102C/T polymorphism in 5-HT2A gene (DOC)

S6 Table Main data of all included studies for the-1438G/A polymorphism in 5-HT2A gene (DOC)

S7 Table Main data of all included studies for the -796 C/G polymorphism in 5-HT2C gene (DOC)

S8 Table Main data of all included studies for the L/S polymorphism in 5-HTTLPR gene (DOC)

S9 Table Main data of all included studies for the 10/12 polymorphism in 5-HTTVNTR gene (DOC)

S10 Table Main data of all included studies for the Gln223Arg polymorphism in LEPR gene (DOC)

S11 Table Main data of all included studies for the Pro12Ala polymorphism in PPAR-γ gene (DOC)

S12 Table Main data of all included studies for the carrier allele polymorphism in APOE gene (DOC)

S13 Table Main data of all included studies for the Arg389Gly polymorphism in ADRB1 gene (DOC)

S14 Table Main data of all included studies for the Arg16Gly polymorphism in ADRB2 gene (DOC)
