# Supplementary figures and images for: Obstructive Sleep Apnea Susceptibility Genes in Chinese Population: A Field Synopsis and Meta-Analysis of Genetic Association Studies
Source: PLoS One. 2015 Aug 18;10(8):e0135942. doi: 10.1371/journal.pone.0135942 (PMC4540430; doi:10.1371/journal.pone.0135942)

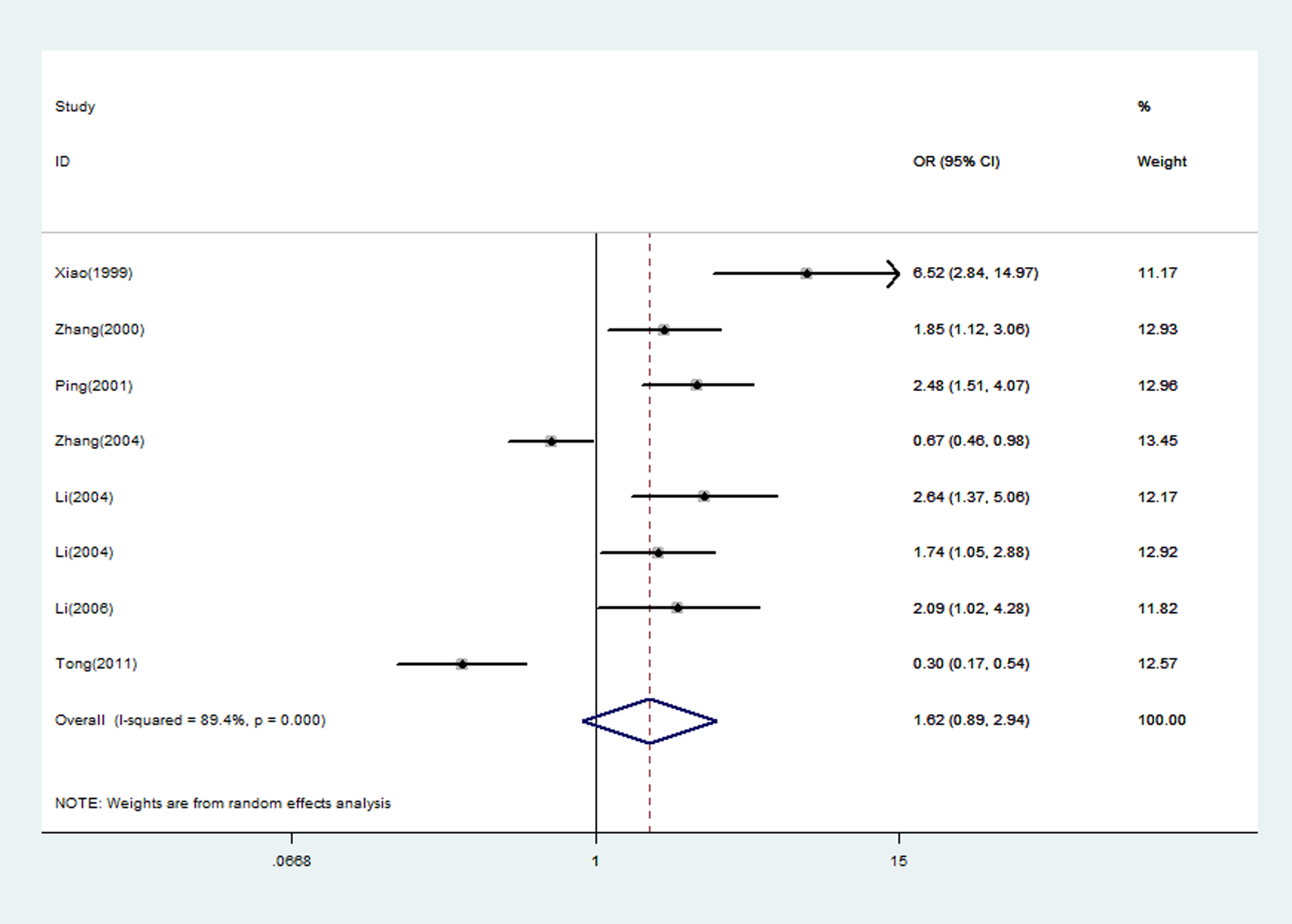

Supplement: S1 Fig — (TIF) [file pone.0135942.s002.tif]

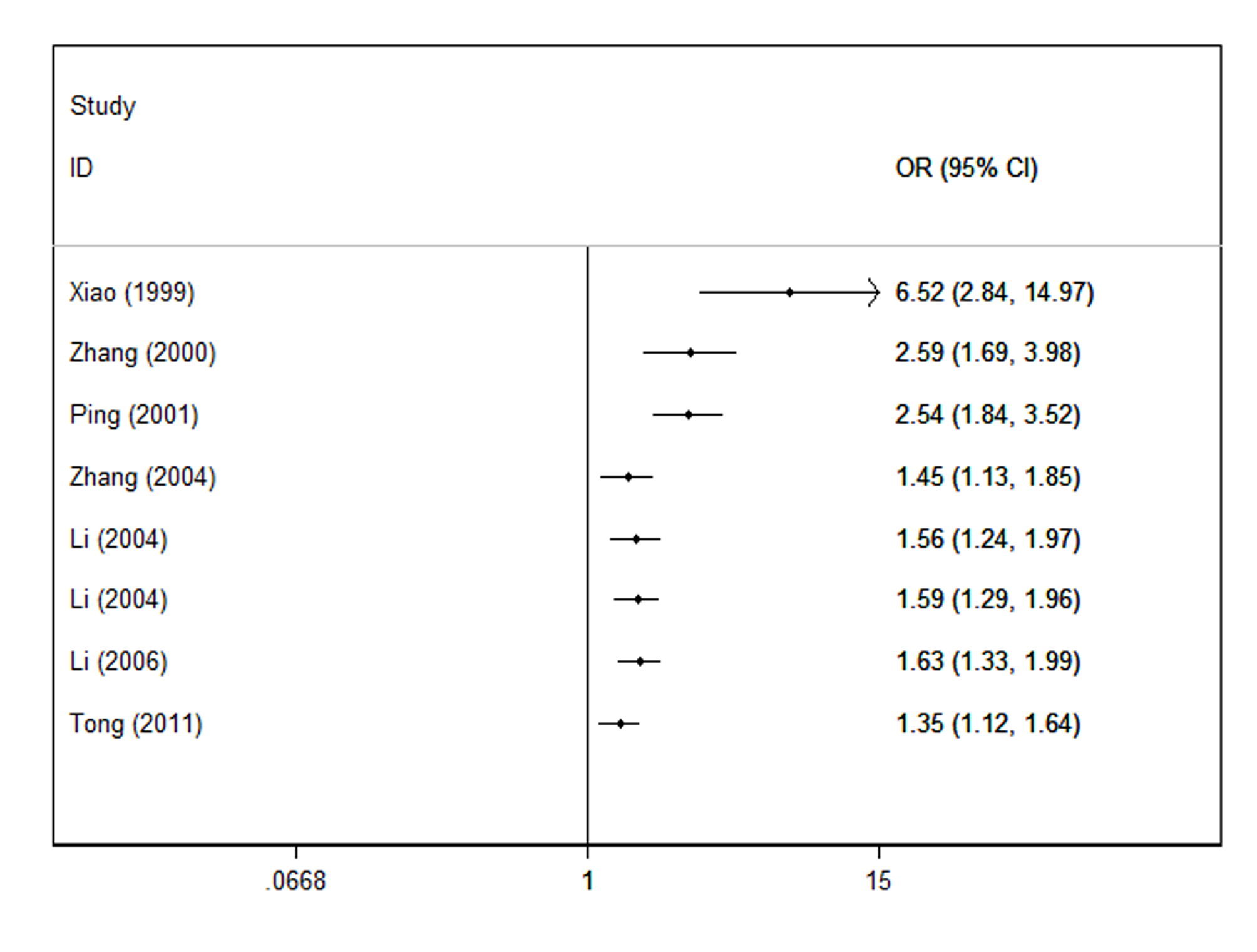

Supplement: S2 Fig — The pooled odds ratio with the corresponding 95% confidence interval at the end of each year-information step is shown. (TIF) [file pone.0135942.s003.tif]

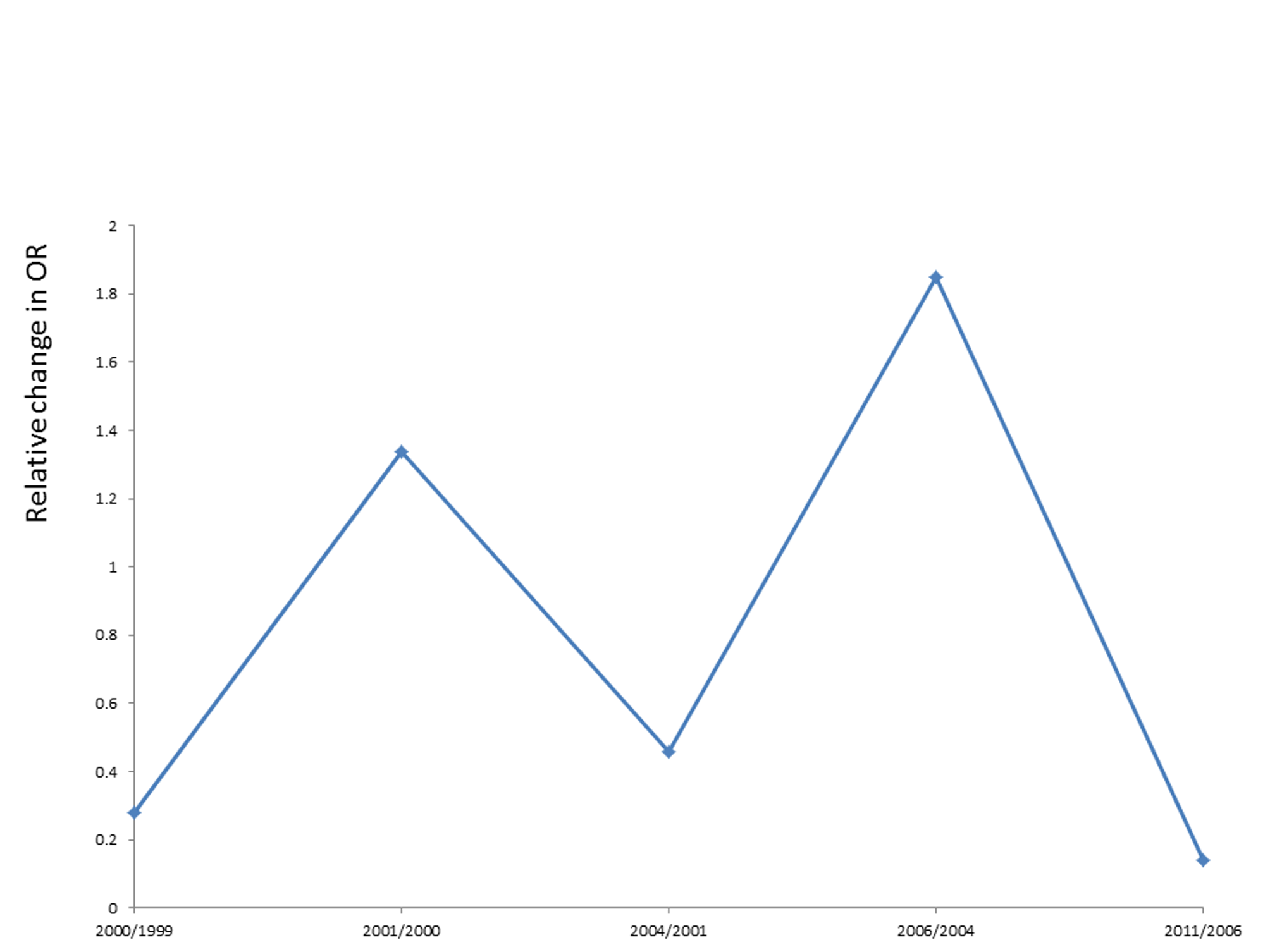

Supplement: S3 Fig — The relative change in the random effects pooled odds ratio (OR) in each information step (OR in next year/OR in current year) is shown. The Y axis represents relative change in OR, and the X axis represents OR in next year/current year. (TIF) [file pone.0135942.s004.tif]

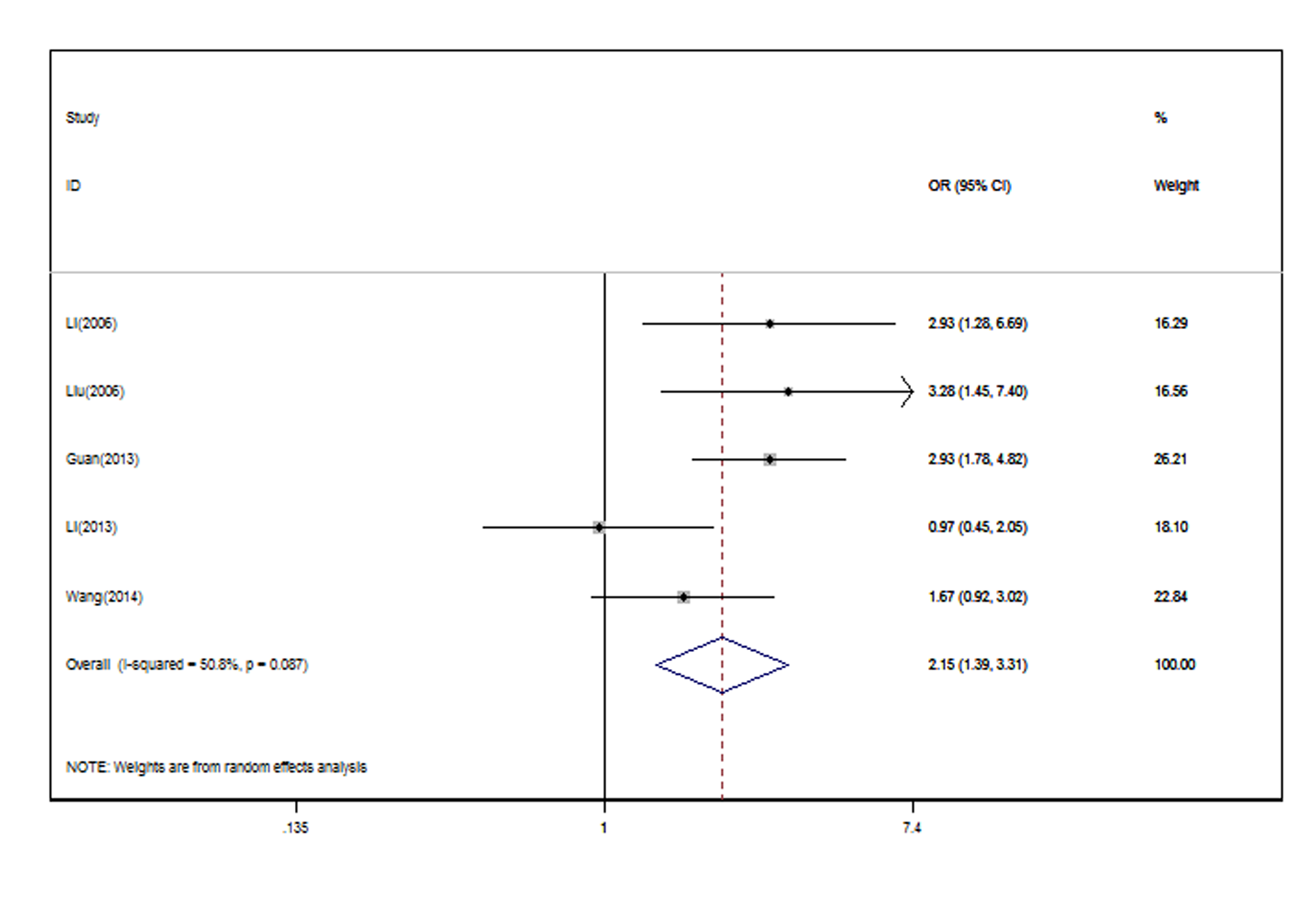

Supplement: S4 Fig — (TIF) [file pone.0135942.s005.tif]

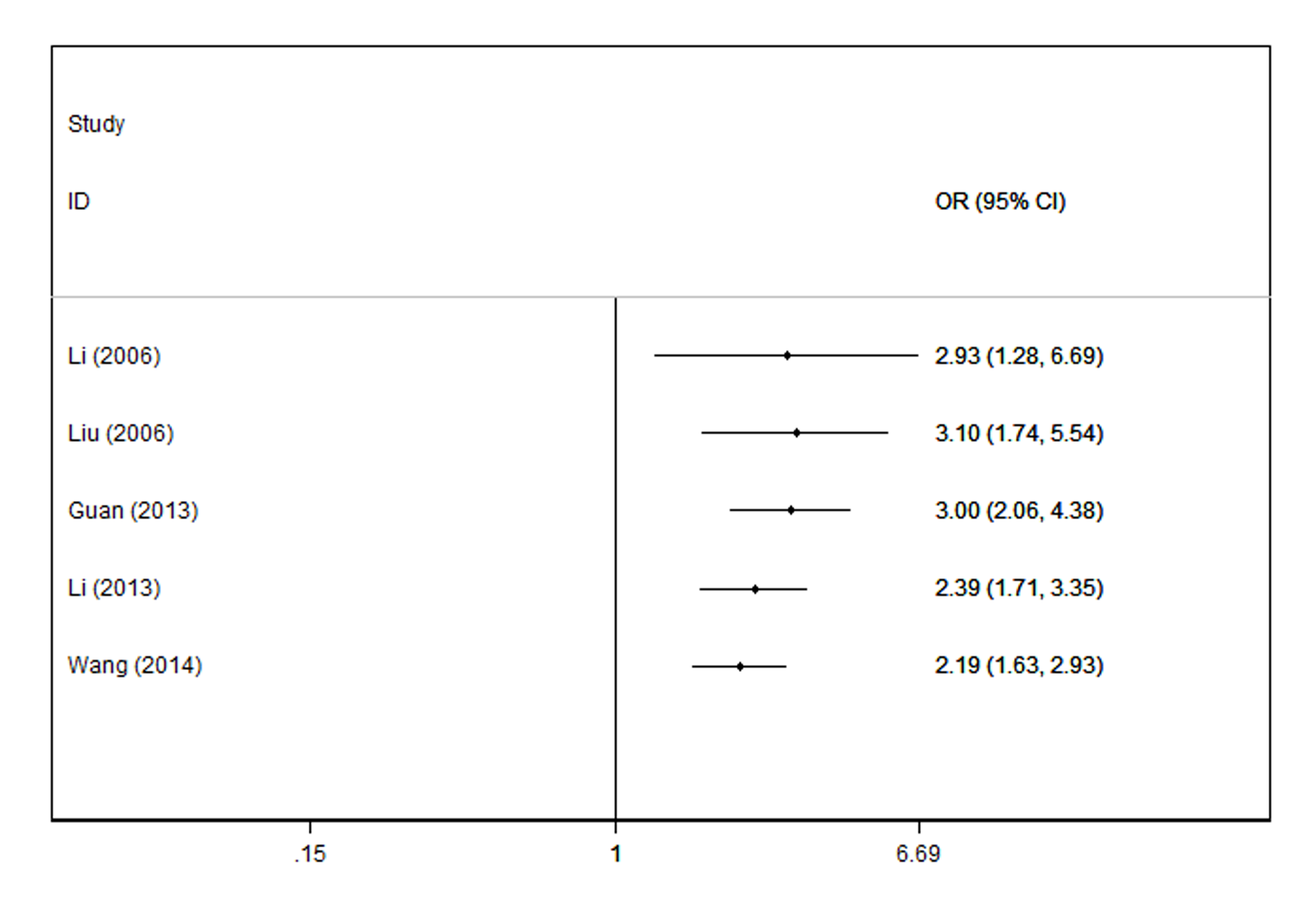

Supplement: S5 Fig — The pooled odds ratio with the corresponding 95% confidence interval at the end of each year-information step is shown. (TIF) [file pone.0135942.s006.tif]

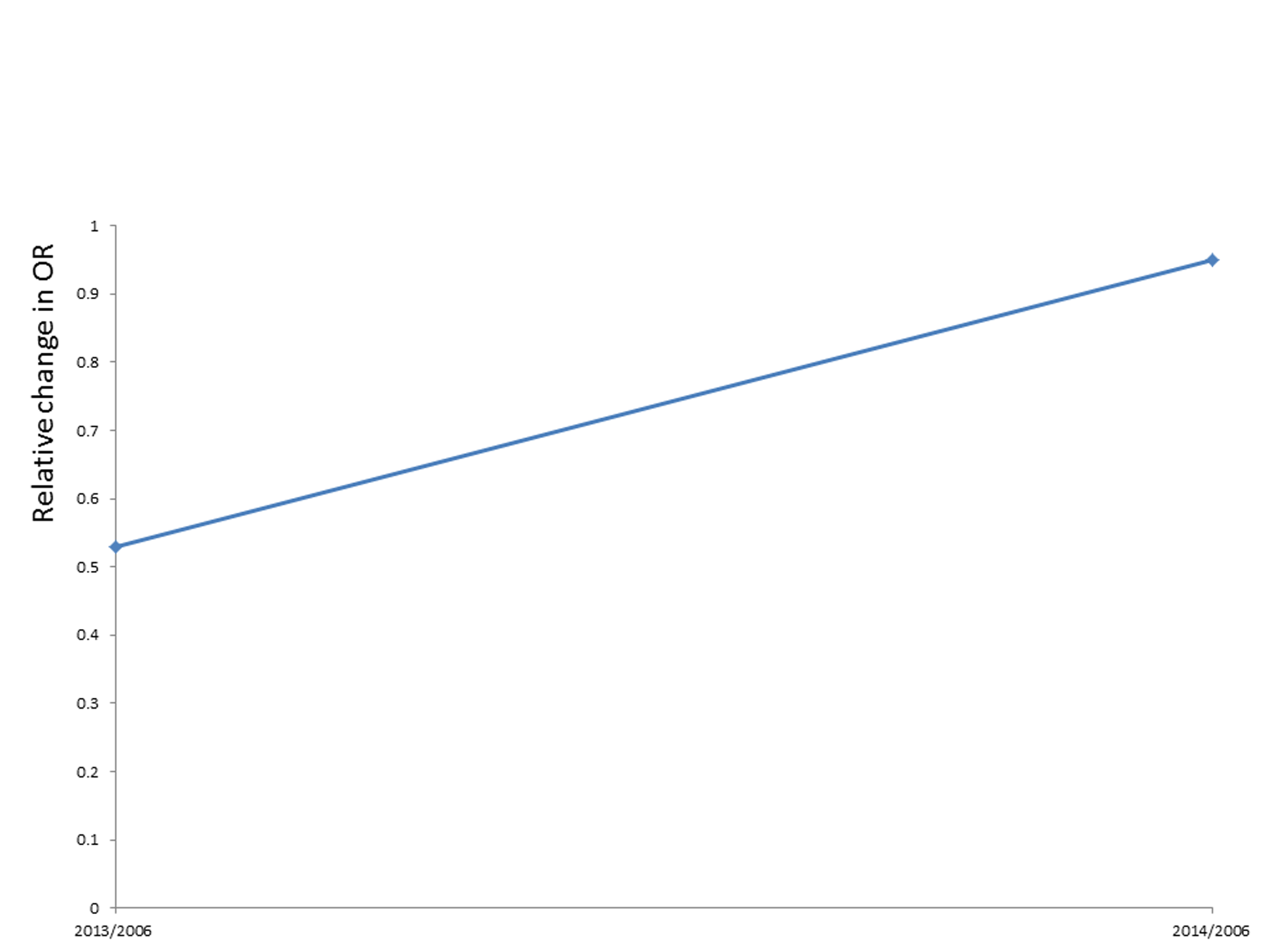

Supplement: S6 Fig — The relative change in the random effects pooled odds ratio (OR) in each information step (OR in next year/OR in current year) is shown. The Y axis represents relative change in OR, and the X axis represents OR in next year/current year. (TIF) [file pone.0135942.s007.tif]

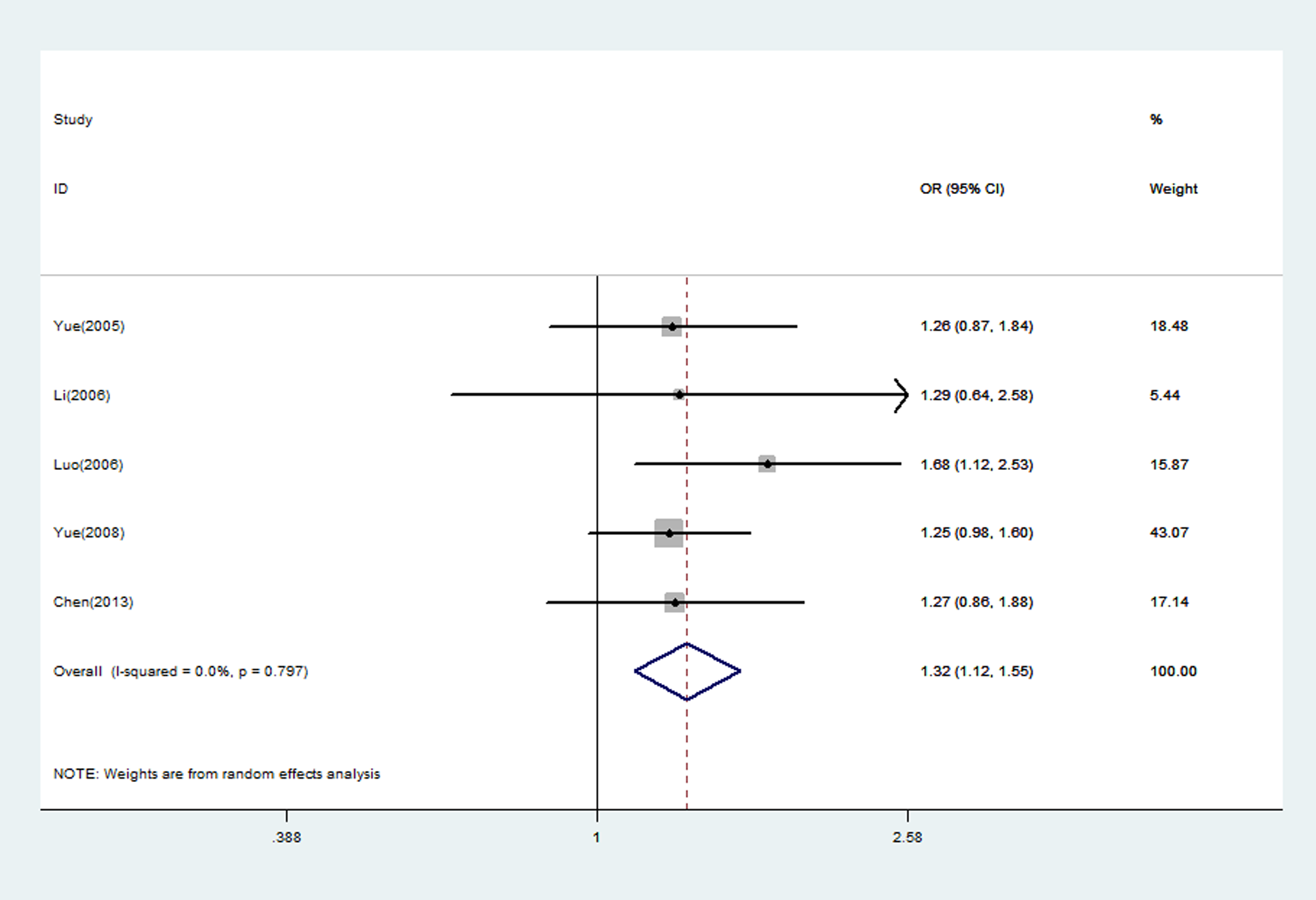

Supplement: S7 Fig — (TIF) [file pone.0135942.s008.tif]

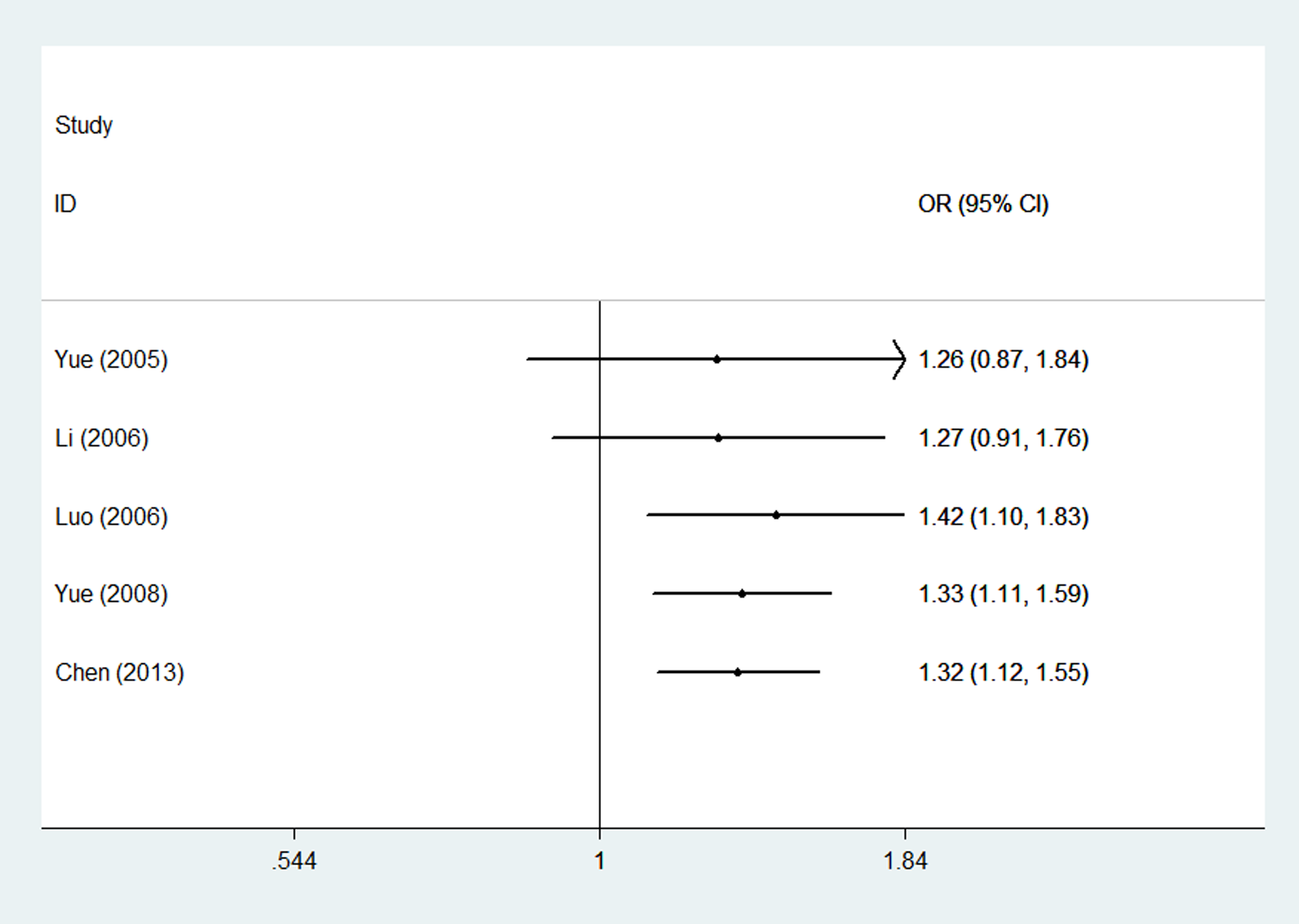

Supplement: S8 Fig — The pooled odds ratio with the corresponding 95% confidence interval at the end of each year-information step is shown. (TIF) [file pone.0135942.s009.tif]

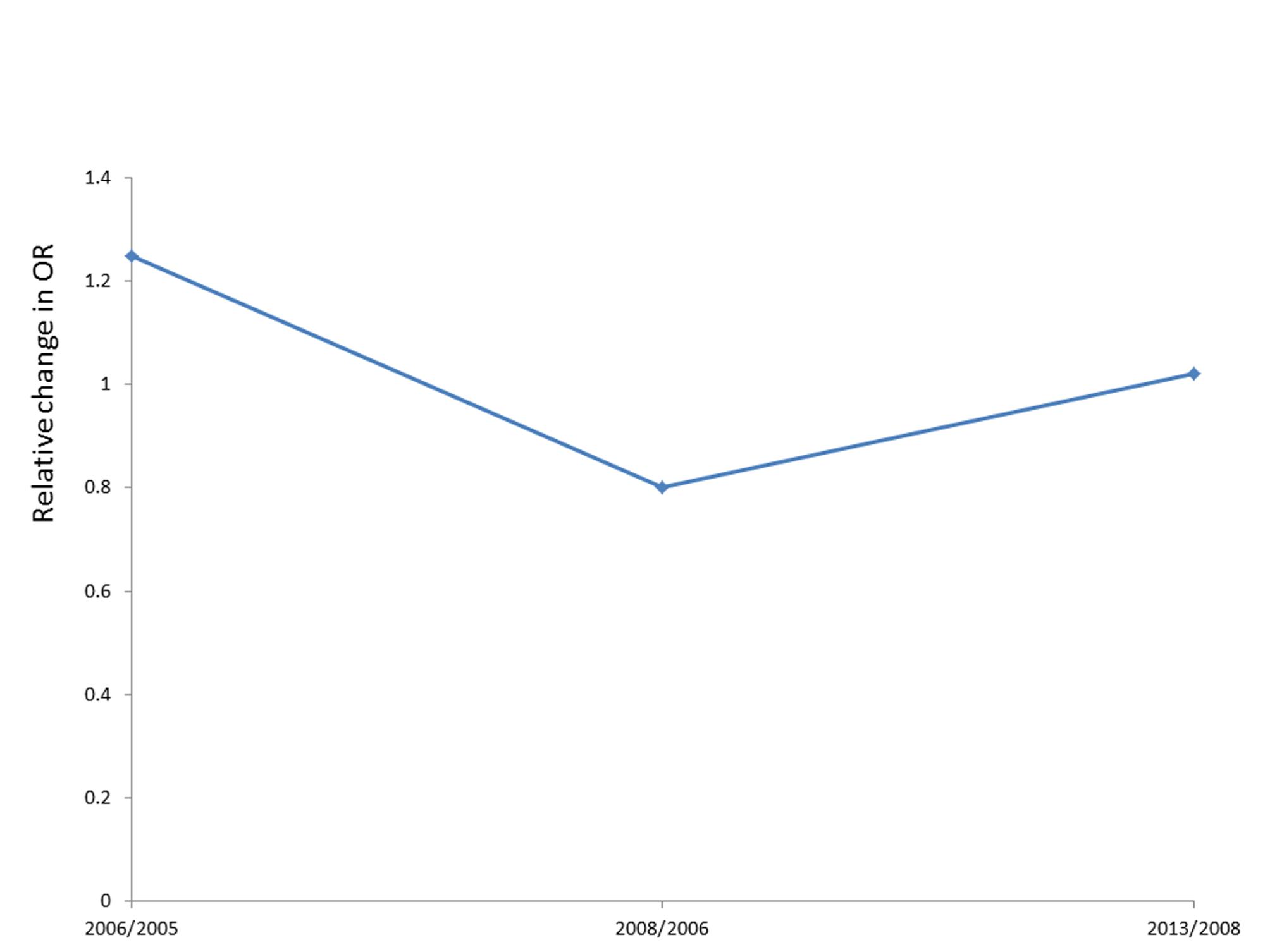

Supplement: S9 Fig — The relative change in the random effects pooled odds ratio (OR) in each information step (OR in next year/OR in current year) is shown. The Y axis represents relative change in OR, and the X axis represents OR in next year/current year. (TIF) [file pone.0135942.s010.tif]
