# Supplementary material for: Obstructive Sleep Apnea Susceptibility Genes in Chinese Population: A Field Synopsis and Meta-Analysis of Genetic Association Studies
Source: PLoS One. 2015 Aug 18;10(8):e0135942. doi: 10.1371/journal.pone.0135942 (PMC4540430; doi:10.1371/journal.pone.0135942)
Supplement: S1 Table — (DOC) [file pone.0135942.s011.doc]

S1 Table. Characteristics of included studies in the meta-analysis

| Author (year) | OSA subjects | | | | |  | Control subjects | | | |
| --- | --- | --- | --- | --- | --- | --- | --- | --- | --- | --- |
|  | Age | BMI | Gender  (M/F) | AHI  (events/h) |  | | Age  (year) | BMI  (Kg/m^2^) | Gender  (M/F) | AHI  (events/h) |
| **ACE I/D**  Xiao(1999) | 50.9±10.5 | NR | 45/5 | NR |  | | 30.9±6.6 | NR | 30/20 | NR |
| Zhang(2000) | NR | NR | 73/40 | ≥5 |  | | NR | NR | 52/9 | <5 |
| Ping(2001) | 60 | NR | 65/15 | ≥5 |  | | 62 | NR | 38/22 | <5 |
| Zhang(2004) | 43.2±2.3 | 28.8±1.4 | NR | ≥5 |  | | 40.1±2.1 | 26.2±2 | NR | <5 |
| Li(2004) | 45±11 | 28.97±3.8 | 77/18 | ≥5 |  | | 45± 13 | 25±4.6 | 39/11 | <5 |
| Li(2004) | NR | NR | 56/4 | ≥5 |  | | 45 | 27 | 26/4 | <5 |
| Li(2006) | NR | NR | 57/8 | ≥5 |  | | 45± 13 | 23.2± 2.5 | 18/2 | <5 |
| Tong(2011) | 58.6±9.8 | 25.7±10.4 | 42/9 | ≥5 |  | | 56.6±5.5 | 25.3±2.4 | 32/28 | 5.2±4.9 |
| **TNF-α-308A/G** |  |  |  |  |  | |  |  |  |  |
| Li(2006) | 39.7±7.9 | 30.6±4.6 | 22/2 | ≥5 |  | | 38.3±9.2 | 29.7±4.6 | 42/6 | <5 |
| Liu(2006) | 44.3±9.8 | 26.3±3.5 | 67/9 | ≥5 |  | | 41.7±10.1 | 25.7±3.3 | 37/5 | <5 |
| Guan(2013) | 43.6±11.7 | 27.4±3.4 | 452/79 | 49.9±26.8 |  | | 42.6±13.1 | 26.89±3.7 | 126/36 | <5 |
| Li(2013) | 45.0±9.0 | 29.4±2.1 | 155/0 | 57.5±16.3 |  | | 46.3±8.0 | 24.1±2.3 | 100/0 | <5 |
| Wang(2014) | 45.2±9.7 | 27.1±3.4 | 60/18 | ≥5 |  | | 43.7±9.8 | 25.6±3.5 | 54/22 | <5 |
| **IL-6-572G/C** |  |  |  |  |  | |  |  |  |  |
| Zhang(2009) | 42.5±1.0 | 28.4±0.4 | NR | 55.6±1.8 |  | | 40.1±1.5 | 25.3±0.4 | NR | 6±0.5 |
| Li(2014) | 45.0±9.0 | 29.4±2.1 | 300/0 | 57.5±16.3 |  | | 46.3±8.0 | 24.1±2.3 | 100/0 | <5 |
| **5-HT2A 102C/T** |  |  |  |  |  | |  |  |  |  |
| Zhu(2007) | 44.2±2.0 | NR | NR | ≥5 |  | | 43±2.1 | NR | NR | <5 |
| Yin(2012) | 41.4±9.2 | 25.9±2.2 | 186/24 | 42.3±22.1 |  | | 40.6±10.4 | 25.3±3.4 | 89/16 | 2.3±1.6 |
| Chen(2013) | 43.8±3.0 | 26.2±2.5 | NR | 42.6±14.8 |  | | 43±2.7 | 24.4±2.5 | NR | 3.7±1.3 |
| **5-HT2A-1438 G/A** |  |  |  |  |  | |  |  |  |  |
| Luo(2006) | 39.6±8.8 | NR | 93/0 | ≥5 |  | | 42±10 | NR | 115/0 | <5 |
| Zhu(2007) | 44.2±2.0 | NR | NR | ≥5 |  | | 43±2.1 | NR | NR | <5 |
| Yin(2012) | 41.4±9.2 | 25.9±2.2 | 186/24 | 42.3±22.1 |  | | 40.6±10.4 | 25.3±3.4 | 89/16 | 2.3±1.6 |
| Chen(2013) | 43.8±3.0 | 26.2±2.5 | NR | 42.6±14.8 |  | | 43±2.7 | 24.4±2.5 | NR | 3.7±1.3 |
| **5-HT2C-796 C/G** |  |  |  |  |  | |  |  |  |  |
| Zhu(2007) | 44.2±2.0 | NR | NR | ≥5 |  | | 43±2.1 | NR | NR | <5 |
| Chen(2013) | 43.8±3.0 | 26.2±2.5 | NR | 42.6±14.8 |  | | 43±2.7 | 24.4±2.5 | NR | 3.7±1.3 |
| **5-HTTLPR L/S** |  |  |  |  |  | |  |  |  |  |
| Yue(2005) | 42.3±10.1 | 25.2±6.3 | 87/17 | ≥5 |  | | NR | 21.3±7.5 | NR | <5 |
| Li(2006) | 39.7±7.9 | 30.6±4.6 | 22/2 | ≥5 |  | | 38.3±9.2 | 29.7±4.6 | 42/6 | <5 |
| Luo(2006) | 39.6±8.8 | NR | 93/0 | ≥5 |  | | 42±10 | NR | 115/0 | <5 |
| Yue(2008) | 45.2±11.8 | 25.1±6.5 | 220/34 | 53.9±16.4 |  | | 43.2±12.7 | 22.6±5.7 | 291/47 | <5 |
| Chen(2013) | 43.8±3.0 | 26.2±2.5 | NR | 42.6±14.8 |  | | 43±2.7 | 24.4±2.5 | NR | 3.7±1.3 |
| **5-HTTVNTR10/12** |  |  |  |  |  | |  |  |  |  |
| Yue(2005) | 42.3±10.1 | 25.2±6.3 | 87/17 | ≥5 |  | | NR | 21.3±7.5 | NR | <5 |
| Luo(2006) | 39.6±8.8 | NR | 93/0 | ≥5 |  | | 42±10 | NR | 115/0 | <5 |
| Yue(2008) | 45.2±11.8 | 25.1±6.5 | 220/34 | 53.9±16.4 |  | | 43.2±12.7 | 22.6±5.7 | 291/47 | <5 |
| Chen(2013) | 43.8±3.0 | 26.2±2.5 | NR | 42.6±14.8 |  | | 43±2.7 | 24.4±2.5 | NR | 3.7±1.3 |
| **LEPR Gln223Arg** |  |  |  |  |  | |  |  |  |  |
| Huang(2003) | 43.4±0.8 | 28.6±0.4 | 100/3 | ≥5 |  | | 43.9±1.2 | 25.8±0.4 | 75/3 | <5 |
| Han(2012) | 46.1±8.1 | NR | 183/0 | ≥5 |  | | NR | NR | 201/0 | <5 |
| Li(2014) | NR | 29.3±0.7 | NR | 29.9±1.97 |  | | NR | 23.98±0.3 | NR | 4.8±0.3 |
| **PPAR-γPro12Ala** |  |  |  |  |  | |  |  |  |  |
| Du (2010) | NR | NR | 100/0 | NR |  | | NR | NR | 100/0 | NR |
| Guan(2011) | 44.2±13.4 | 27.2±3.5 | 357/63 | ≥5 |  | | 44.5±10.6 | 26.9±3.7 | 151/39 | <5 |
| **APOE** |  |  |  |  |  | |  |  |  |  |
| Zheng(2007) | 39.0±7.4 | NR | 50/0 | ≥5 |  | | 44.5±10.3 | NR | 40/0 | <5 |
| Sheng(2008) | 48.6±11.6 | 29.6±4.0 | 73/11 | ≥5 |  | | 49.8±11.9 | 24.7±2.8 | 92/14 | <5 |
| **ADRB1 Arg389Gly** |  |  |  |  |  | |  |  |  |  |
| Luo(2008) | NR | NR | NR | ≥5 |  | | NR | NR | NR | <5 |
| Wang(2008) | 47±13 | 29.4±2.8 | 164/28 | ≥5 |  | | NR | NR | NR | <5 |
| **ADRB2 Arg16Gly** |  |  |  |  |  | |  |  |  |  |
| Zhang(2005) | 43.6±2.5 | 27.4±1.72 | 165/0 | 43.4±14.2 |  | | 42.5±2.2 | 26.7±1.66 | 153/0 | 2±0.7 |
| Luo(2008) | NR | NR | NR | ≥5 |  | | NR | NR | NR | <5 |

Abbreviation: NR, not reported; OSA, obstructive sleep apnea; NR, not reported; BMI, body mass index; M, male; F, female; AHI, apnea-hypopnea index; ACE, angiotensin-converting enzyme; I/D, insertion/deletion; TNF, tumor necrosis factor; IL-6, Interleukin-6; 5-HTR, 5-hydroxytryptamine receptor; 5-HTT, 5-hydroxytryptamine transporter; LPR, linked promoter region; VNTR, variable number tandem repeat; PPAR-γ, peroxisome proliferator-activated receptor; APOE, apolipoprotein E; ADRB, β-adrenergic receptor.
