# Supplementary material for: Obstructive Sleep Apnea Susceptibility Genes in Chinese Population: A Field Synopsis and Meta-Analysis of Genetic Association Studies
Source: PLoS One. 2015 Aug 18;10(8):e0135942. doi: 10.1371/journal.pone.0135942 (PMC4540430; doi:10.1371/journal.pone.0135942)
Supplement: S2 Table — (DOC) [file pone.0135942.s012.doc]

S2 Table. Main data of all included studies for the I/D polymorphism in ACE gene

| Author (year) | Ethnicity | Age | Genotyping method | HWE | Cases/Controls | OSA | | | Control | | | OR_G_(95%CI) |
| --- | --- | --- | --- | --- | --- | --- | --- | --- | --- | --- | --- | --- |
|  |  |  |  |  |  | II | ID | DD | II | ID | DD |  |
| Xiao(1999) | Han | NR | PCR | 0.63 | 50/50 | 22 | 28 | 0 | 16 | 26 | 8 | 2.07(0.99-4.34) |
| Zhang(2000) | Han | NR | PCR | 0.46 | 61/68 | 30 | 20 | 11 | 19 | 31 | 18 | 1.97(1.09-3.55) |
| Ping(2001) | Han | 60 | PCR | 0.99 | 80/60 | 42 | 30 | 8 | 15 | 30 | 15 | 2.89(1.59-5.27) |
| Zhang(2004) | Han | 43.2±2.3 | PCR | 0.11 | 121/100 | 39 | 46 | 36 | 41 | 40 | 19 | 0.66(0.43-1.03) |
| Li(2004) | Han | 44.6±8.7 | PCR | <0.01 | 60/30 | 27 | 12 | 21 | 8 | 3 | 19 | 2.41(1.21-4.80) |
| Li(2004) | Han | 45.4±11.5 | PCR | <0.01 | 92/50 | 8 | 71 | 13 | 0 | 34 | 16 | 2.42(1.19-4.92) |
| Li(2006) | NR | 45±11 | PCR | <0.01 | 65/20 | 39 | 4 | 22 | 7 | 4 | 9 | 2.00(1.02-3.92) |
| Tong(2011) | Han | 58.6±9.8 | PCR | 0.12 | 51/60 | 6 | 13 | 32 | 13 | 36 | 11 | 0.24(0.12-0.50) |

Abbreviation: NR, not reported; OR_G_, generalized odds ratio; CI, confidential interval; ACE, angiotensin-converting enzyme; I/D, insertion/deletion; PCR, polymerase chain reaction; HWE, Hardy-Weinberg equilibrium.
