# Supplementary material for: Obstructive Sleep Apnea Susceptibility Genes in Chinese Population: A Field Synopsis and Meta-Analysis of Genetic Association Studies
Source: PLoS One. 2015 Aug 18;10(8):e0135942. doi: 10.1371/journal.pone.0135942 (PMC4540430; doi:10.1371/journal.pone.0135942)
Supplement: S3 Table — (DOC) [file pone.0135942.s013.doc]

S3 Table. Main data of all included studies for the -308A/G polymorphism in TNF-α gene

| Author (year) | Ethnicity | Age | Genotyping method | HWE | Cases/Controls | OSA | | | Control | | | OR_G_ (95%CI) |
| --- | --- | --- | --- | --- | --- | --- | --- | --- | --- | --- | --- | --- |
|  |  |  |  |  |  | GG | AG | AA | GG | AG | AA |  |
| Li(2006) | Han | 39.7±7.9 | PCR | 0.98 | 24/48 | 11 | 10 | 3 | 35 | 12 | 1 | 3.21(1.22-8.45) |
| Liu(2006) | Han | 44.3±9.8 | PCR-RFLP | 0.07 | 76/42 | 45 | 23 | 8 | 35 | 6 | 1 | 3.35(1.37-8.23) |
| Guan(2013) | Han | 43.6±11.7 | PCR-RFLP | 0.60 | 531/162 | 430 | 95 | 6 | 143 | 18 | 1 | 1.76(1.04-2.97) |
| Li(2013) | Han | 45.0±9.0 | PCR | 0.52 | 155/100 | 137 | 18 | 0 | 88 | 12 | 0 | 0.95(0.44-2.04) |
| Wang(2014) | Uygur | 45.2±9.7 | PCR | 0.06 | 78/78 | 54 | 16 | 8 | 66 | 10 | 2 | 1.18(1.14-1.21) |

Abbreviation: OR_G_, generalized odds ratio; CI, confidential interval; TNF-α, tumor necrosis factor-α; PCR, polymerase chain reaction; HWE, Hardy-Weinberg equilibrium; PCR-RFLP, PCR-restriction fragment length polymorphism.
