# Supplementary material for: Obstructive Sleep Apnea Susceptibility Genes in Chinese Population: A Field Synopsis and Meta-Analysis of Genetic Association Studies
Source: PLoS One. 2015 Aug 18;10(8):e0135942. doi: 10.1371/journal.pone.0135942 (PMC4540430; doi:10.1371/journal.pone.0135942)
Supplement: S4 Table — (DOC) [file pone.0135942.s014.doc]

S4 Table. Main data of all included studies for the -572G/C polymorphism in IL-6 gene

| Author (year) | Ethnicity | Age | Genotyping method | HWE | Cases/Controls | OSA | | | Control | | | OR_G_(95%CI) |
| --- | --- | --- | --- | --- | --- | --- | --- | --- | --- | --- | --- | --- |
|  |  |  |  |  |  | GG | GC | CC | GG | GC | CC |  |
| Zhang(2009) | Han | 42.5±1.0 | PCR-RFLP | 0.20 | 151/75 | 11 | 61 | 79 | 5 | 37 | 33 | 0.77(0.46-1.27) |
| Li(2014) | Han | 45.0±9.0 | PCR-RFLP | 0.36 | 300/100 | 23 | 125 | 152 | 5 | 42 | 53 | 1.13(0.75-1.72) |

Abbreviation: OR_G_, generalized odds ratio; CI, confidential interval; IL-6, interleukin-6; tumor necrosis factor-α; HWE, Hardy-Weinberg equilibrium; PCR-RFLP, polymerase chain reaction-restriction fragment length polymorphism.
