# Supplementary material for: Obstructive Sleep Apnea Susceptibility Genes in Chinese Population: A Field Synopsis and Meta-Analysis of Genetic Association Studies
Source: PLoS One. 2015 Aug 18;10(8):e0135942. doi: 10.1371/journal.pone.0135942 (PMC4540430; doi:10.1371/journal.pone.0135942)
Supplement: S5 Table — (DOC) [file pone.0135942.s015.doc]

S5 Table. Main data of all included studies for the -102C/T polymorphism in 5-HTR2A gene

| Author (year) | Ethnicity | Age | Genotyping method | HWE | Cases/Controls | OSA | | | Control | | | OR_G_(95%CI) |
| --- | --- | --- | --- | --- | --- | --- | --- | --- | --- | --- | --- | --- |
|  |  |  |  |  |  | TT | TC | CC | TT | TC | CC |  |
| Zhu(2007) | Han | 44.2±2.0 | PCR | 0.75 | 65/54 | 18 | 30 | 17 | 15 | 28 | 11 | 0.87(0.48-1.60) |
| Yin(2012) | Han | 41.4±9.2 | PCR | 0.79 | 210/105 | 52 | 107 | 51 | 30 | 51 | 24 | 0.88(0.59-1.31) |
| Chen(2013) | Han | 43.8±3.0 | PCR-RFLP | 0.73 | 121/105 | 34 | 56 | 31 | 29 | 54 | 22 | 0.91(0.59-1.41) |

Abbreviation: OR_G_, generalized odds ratio; CI, confidential interval; 5-HTR2A, 5-hydroxytryptamine receptor (5-HTR) 2A; PCR, polymerase chain reaction; HWE, Hardy-Weinberg equilibrium; PCR-RFLP, PCR-restriction fragment length polymorphism.
