# Supplementary material for: Obstructive Sleep Apnea Susceptibility Genes in Chinese Population: A Field Synopsis and Meta-Analysis of Genetic Association Studies
Source: PLoS One. 2015 Aug 18;10(8):e0135942. doi: 10.1371/journal.pone.0135942 (PMC4540430; doi:10.1371/journal.pone.0135942)
Supplement: S6 Table — (DOC) [file pone.0135942.s016.doc]

S6 Table. Main data of all included studies for the-1438G/A polymorphism in 5-HTR2A gene

| Author (year) | Ethnicity | Age | Genotyping method | HWE | Cases/Controls | OSA | | | Control | | | OR_G_(95%CI) |
| --- | --- | --- | --- | --- | --- | --- | --- | --- | --- | --- | --- | --- |
|  |  |  |  |  |  | AA | AG | GG | AA | AG | GG |  |
| Luo(2006) | Han | 39.6±8.8 | PCR | 0.96 | 93/115 | 26 | 49 | 18 | 51 | 51 | 13 | 0.53(0.33-0.86) |
| Zhu(2007) | Han | 44.2±2.0 | PCR | <0.01 | 65/54 | 41 | 15 | 9 | 15 | 14 | 25 | 1.97(0.99-4.34) |
| Yin(2012) | Han | 41.4±9.2 | PCR | <0.01 | 210/105 | 85 | 82 | 43 | 17 | 67 | 21 | 2.89(1.59-5.27) |
| Chen(2013) | Han | 43.8±3.0 | PCR-RFLP | <0.01 | 121/105 | 74 | 30 | 17 | 28 | 28 | 49 | 0.66(0.43-1.03) |

Abbreviation: OR_G_, generalized odds ratio; CI, confidential interval; 5-HTR2A, 5-hydroxytryptamine receptor (5-HTR) 2A; PCR, polymerase chain reaction; HWE, Hardy-Weinberg equilibrium; PCR-RFLP, PCR-restriction fragment length polymorphism.
