# Supplementary material for: Obstructive Sleep Apnea Susceptibility Genes in Chinese Population: A Field Synopsis and Meta-Analysis of Genetic Association Studies
Source: PLoS One. 2015 Aug 18;10(8):e0135942. doi: 10.1371/journal.pone.0135942 (PMC4540430; doi:10.1371/journal.pone.0135942)
Supplement: S7 Table — (DOC) [file pone.0135942.s017.doc]

S7 Table. Main data of all included studies for the -796 C/G polymorphism in 5-HTR2C gene

| Author (year) | Ethnicity | Age | Genotyping method | HWE | Cases/Controls | OSA | | | Control | | | OR_G_(95%CI) |
| --- | --- | --- | --- | --- | --- | --- | --- | --- | --- | --- | --- | --- |
|  |  |  |  |  |  | GG | GC | CC | GG | GC | CC |  |
| Zhu(2007) | Han | 44.2±2.0 | PCR | 0.49 | 11/12 | 7 | 4 | 0 | 8 | 4 | 0 | 0.88(0.18-4.27) |
| Chen(2013) | Han | 43.8±3.0 | PCR-RFLP | 0.36 | 22/16 | 14 | 8 | 0 | 10 | 6 | 0 | 1.08(0.30-3.83) |

Abbreviation: OR_G_, generalized odds ratio; CI, confidential interval; 5-HTR2C, 5-hydroxytryptamine receptor (5-HTR) 2C; PCR, polymerase chain reaction; HWE, Hardy-Weinberg equilibrium; PCR-RFLP, PCR-restriction fragment length polymorphism.
