# Supplementary material for: Obstructive Sleep Apnea Susceptibility Genes in Chinese Population: A Field Synopsis and Meta-Analysis of Genetic Association Studies
Source: PLoS One. 2015 Aug 18;10(8):e0135942. doi: 10.1371/journal.pone.0135942 (PMC4540430; doi:10.1371/journal.pone.0135942)
Supplement: S8 Table — (DOC) [file pone.0135942.s018.doc]

S8 Table. Main data of all included studies for the L/S polymorphism in 5-HTTLPR gene

| Author (year) | Ethnicity | Age | Genotyping method | HWE | Cases/Controls | OSA | | | Control | | | OR_G_(95%CI) |
| --- | --- | --- | --- | --- | --- | --- | --- | --- | --- | --- | --- | --- |
|  |  |  |  |  |  | L/L | L/S | S/S | L/L | L/S | S/S |  |
| Yue(2005) | Han | 42.3±10.1 | PCR | 0.08 | 104/150 | 20 | 33 | 51 | 18 | 54 | 78 | 1.22(0.79-1.89) |
| Li(2006) | Han | 39.7±7.9 | PCR | 0.98 | 24/48 | 5 | 14 | 5 | 13 | 16 | 19 | 1.35(0.63-2.88) |
| Luo(2006) | Han | 39.6±8.8 | PCR | 0.98 | 93/115 | 21 | 35 | 37 | 10 | 48 | 57 | 1.68(1.04-2.71) |
| Yue(2008) | Han | 45.2±11.8 | PCR | 0.22 | 254/338 | 34 | 106 | 114 | 34 | 131 | 173 | 1.28(0.79-1.93) |
| Chen(2013) | Han | 43.8±3.0 | PCR-RFLP | 0.01 | 121/105 | 27 | 35 | 59 | 16 | 34 | 55 | 1.18(0.76-1.85) |

Abbreviation: OR_G_, generalized odds ratio; CI, confidential interval; 5-HTTLPR, 5-hydroxytryptamine transporter gene-linked promoter region; PCR, polymerase chain reaction; HWE, Hardy-Weinberg equilibrium; PCR-RFLP, PCR-restriction fragment length polymorphism.
