# Supplementary material for: Obstructive Sleep Apnea Susceptibility Genes in Chinese Population: A Field Synopsis and Meta-Analysis of Genetic Association Studies
Source: PLoS One. 2015 Aug 18;10(8):e0135942. doi: 10.1371/journal.pone.0135942 (PMC4540430; doi:10.1371/journal.pone.0135942)
Supplement: S9 Table — (DOC) [file pone.0135942.s019.doc]

S9 Table. Main data of all included studies for the 10/12 polymorphism in 5-HTTVNTR gene

| Author (year) | Ethnicity | Age | Genotyping method | HWE | Cases/Controls | OSA | | | Control | | | OR_G_(95%CI) |
| --- | --- | --- | --- | --- | --- | --- | --- | --- | --- | --- | --- | --- |
|  |  |  |  |  |  | 10/10 | 10/12 | 12/12 | 10/10 | 10/12 | 12/12 |  |
| Yue(2005) | Han | 42.3±10.1 | PCR | 0.35 | 104/150 | 3 | 20 | 81 | 1 | 14 | 135 | 2.55(1.27-5.12) |
| Luo(2006) | Han | 39.6±8.8 | PCR | 0.80 | 93/115 | 0 | 17 | 76 | 1 | 22 | 92 | 0.92(0.46-1.83) |
| Yue(2008) | Han | 45.2±11.8 | PCR | 0.25 | 254/338 | 6 | 46 | 202 | 3 | 41 | 294 | 1.72(1.12-2.65) |
| Chen(2013) | Han | 43.8±3.0 | PCR-RFLP | 0.23 | 121/105 | 8 | 24 | 89 | 1 | 10 | 94 | 3.08(1.49-6.37) |

Abbreviation: OR_G_, generalized odds ratio; CI, confidential interval; 5-HTTVNTR, 5-hydroxytryptamine transporter variable number tandem repeat; PCR, polymerase chain reaction; HWE, Hardy-Weinberg equilibrium; PCR-RFLP, PCR-restriction fragment length polymorphism.
