# Supplementary material for: Obstructive Sleep Apnea Susceptibility Genes in Chinese Population: A Field Synopsis and Meta-Analysis of Genetic Association Studies
Source: PLoS One. 2015 Aug 18;10(8):e0135942. doi: 10.1371/journal.pone.0135942 (PMC4540430; doi:10.1371/journal.pone.0135942)
Supplement: S10 Table — (DOC) [file pone.0135942.s020.doc]

S10 Table. Main data of all included studies for the Gln223Arg polymorphism in LEPR gene

| Author (year) | Ethnicity | Age | Genotyping method | HWE | Cases/Controls | OSA | | | Control | | | OR_G_(95%CI) |
| --- | --- | --- | --- | --- | --- | --- | --- | --- | --- | --- | --- | --- |
|  |  |  |  |  |  | GG | GA | AA | GG | GA | AA |  |
| Huang(2003) | Han | 43.4±0.8 | PCR | 0.72 | 103/78 | 79 | 22 | 2 | 64 | 13 | 1 | 0.72(0.35-1.49) |
| Han(2012) | NR | 46.1±8.1 | PCR | 0.94 | 183/201 | 147 | 35 | 1 | 162 | 37 | 2 | 0.99(0.60-1.63) |
| Li(2014) | Han | NR | PCR | 0.44 | 60/60 | 51 | 6 | 3 | 50 | 9 | 1 | 1.09(0.42-2.80) |

Abbreviation: OR_G_, generalized odds ratio; CI, confidential interval; LEPR, leptin receptor; PCR, polymerase chain reaction; HWE, Hardy-Weinberg equilibrium.
