# Supplementary material for: Obstructive Sleep Apnea Susceptibility Genes in Chinese Population: A Field Synopsis and Meta-Analysis of Genetic Association Studies
Source: PLoS One. 2015 Aug 18;10(8):e0135942. doi: 10.1371/journal.pone.0135942 (PMC4540430; doi:10.1371/journal.pone.0135942)
Supplement: S11 Table — (DOC) [file pone.0135942.s021.doc]

S11 Table. Main data of all included studies for the Pro12Ala polymorphism in PPAR-γ gene

| Author (year) | Ethnicity | Age | Genotyping method | HWE | Cases/Controls | OSA | | | Control | | | OR_G_ (95%CI) |
| --- | --- | --- | --- | --- | --- | --- | --- | --- | --- | --- | --- | --- |
|  |  |  |  |  |  | PP | PA | AA | PP | PA | AA |  |
| Du (2010) | NR | NR | PCR | 0.68 | 100/100 | 90 | 10 | 0 | 92 | 8 | 0 | 0.79(0.31-2.04) |
| Guan(2011) | Han | 44.2±13.4 | PCR-RFLP | 0.57 | 420/190 | 372 | 47 | 1 | 175 | 15 | 0 | 0.69(0.38-1.25) |

Abbreviation: NR, not report; OR_G_, generalized odds ratio; CI, confidential interval; TNF-α, tumor necrosis factor-α; PCR, polymerase chain reaction; HWE, Hardy-Weinberg equilibrium; PCR-RFLP, PCR-restriction fragment length polymorphism.
