# Supplementary material for: Obstructive Sleep Apnea Susceptibility Genes in Chinese Population: A Field Synopsis and Meta-Analysis of Genetic Association Studies
Source: PLoS One. 2015 Aug 18;10(8):e0135942. doi: 10.1371/journal.pone.0135942 (PMC4540430; doi:10.1371/journal.pone.0135942)
Supplement: S12 Table — (DOC) [file pone.0135942.s022.doc]

S12 Table. Main data of all included studies for the carrier allele polymorphism in APOE gene

| Author (year) | Ethnicity | Age | Genotyping method | HWE | Cases/Controls | OSA | | | Control | | | OR_G_ (95%CI) |
| --- | --- | --- | --- | --- | --- | --- | --- | --- | --- | --- | --- | --- |
|  |  |  |  |  |  | ε2 | ε3 | ε4 | ε2 | ε3 | ε4 |  |
| Zheng(2007) | NR | 39.0±7.4 | PCR | 0.12 | 50/40 | 13 | 71 | 16 | 9 | 66 | 5 | 2.10 (0.97-4.55), |
| Sheng(2008) | Han | 48.6±11.6 | PCR | 0.56 | 84/106 | 10 | 114 | 44 | 31 | 169 | 12 | 2.28 (1.40-3.73) |

Abbreviation: OR_G_, generalized odds ratio; CI, confidential interval; APOE, apolipoprotein E; PCR, polymerase chain reaction; HWE, Hardy-Weinberg equilibrium.
