# Supplementary material for: Obstructive Sleep Apnea Susceptibility Genes in Chinese Population: A Field Synopsis and Meta-Analysis of Genetic Association Studies
Source: PLoS One. 2015 Aug 18;10(8):e0135942. doi: 10.1371/journal.pone.0135942 (PMC4540430; doi:10.1371/journal.pone.0135942)
Supplement: S13 Table — (DOC) [file pone.0135942.s023.doc]

S13 Table. Main data of all included studies for the Arg389Gly polymorphism in ADRB1 gene

| Author (year) | Ethnicity | Age | Genotyping method | HWE | Cases/Controls | OSA | | | Control | | | OR_G_(95%CI) |
| --- | --- | --- | --- | --- | --- | --- | --- | --- | --- | --- | --- | --- |
|  |  |  |  |  |  | Arg/Arg | Arg/Gly | Gly/Gly | Arg/Arg | Arg/Gly | Gly/Gly |  |
| Luo(2008) | Han | 50.5±8.9 | PCR | 0.7 | 180/36 | 124 | 48 | 8 | 23 | 12 | 1 | 1.20(0.59-2.43) |
| Wang(2008) | Han | 47±13 | PCR | 0.9 | 192/96 | 99 | 76 | 17 | 52 | 37 | 7 | 0.89(0.57-1.40) |

Abbreviation: OR_G_, generalized odds ratio; CI, confidential interval; ADRB1, β1-adrenergic receptor; PCR, polymerase chain reaction; HWE, Hardy-Weinberg equilibrium.
