# Supplementary material for: Obstructive Sleep Apnea Susceptibility Genes in Chinese Population: A Field Synopsis and Meta-Analysis of Genetic Association Studies
Source: PLoS One. 2015 Aug 18;10(8):e0135942. doi: 10.1371/journal.pone.0135942 (PMC4540430; doi:10.1371/journal.pone.0135942)
Supplement: S14 Table — (DOC) [file pone.0135942.s024.doc]

S14 Table. Main data of all included studies for the Arg16Gly polymorphism in ADRB2 gene

| Author (year) | Ethnicity | Age | Genotyping method | HWE | Cases/Controls | OSA | | | Control | | | OR_G_(95%CI) |
| --- | --- | --- | --- | --- | --- | --- | --- | --- | --- | --- | --- | --- |
|  |  |  |  |  |  | Arg/Arg | Arg/Gly | Gly/Gly | Arg/Arg | Arg/Gly | Gly/Gly |  |
| Zhang(2005) | Han | 43.6±2.5 | PCR | 0.94 | 165/153 | 38 | 74 | 53 | 37 | 76 | 40 | 0.84(0.58-1.22) |
| Luo(2008) | Han | 50.5±8.9 | PCR | <0.01 | 180/36 | 124 | 20 | 36 | 25 | 5 | 6 | 1.16(0.65-2.05) |

Abbreviation: OR_G_, generalized odds ratio; CI, confidential interval; ADRB2, β2-adrenergic receptor; PCR, polymerase chain reaction; HWE, Hardy-Weinberg equilibrium.
